# miR-208a-3p suppresses cell apoptosis by targeting PDCD4 in gastric cancer

**DOI:** 10.18632/oncotarget.12006

**Published:** 2016-09-13

**Authors:** Kai Yin, Minghui Liu, Mei Zhang, Feng Wang, Min Fen, Zhijian Liu, Yutao Yuan, Shanting Gao, Liuqing Yang, Weijie Zhang, Jianguo Zhang, Baoliang Guo, Jianwei Xu, Hongwei Liang, Xi Chen, Wenxian Guan

**Affiliations:** ^1^ Department of Gastrointestinal Surgery, Nanjing Drum Tower Hospital Clinical College of Nanjing Medical University, Nanjing, Jiangsu 210008, China; ^2^ State Key Laboratory of Pharmaceutical Biotechnology, NJU Advanced Institute for Life Sciences (NAILS), Jiangsu Engineering Research Center for MicroRNA Biology and Biotechnology, School of Life Sciences, Nanjing University, Nanjing, Jiangsu 210046, China; ^3^ Department of General Surgery, Taixing Hospital Affiliated to Bengbu Medical School, Taixing, Jiangsu 225400, China; ^4^ Department of Anesthesiology, Taixing Hospital Affiliated to Bengbu Medical School, Taixing, Jiangsu 225400, China; ^5^ Department of Gastrointestinal Surgery, Nanjing Drum Tower Hospital, Medical School of Nanjing University, Nanjing, Jiangsu 210008, China; ^6^ Department of General Surgery, The Second Affiliated Hospital of Harbin Medical University, Harbin, Heilongjiang 150081, China

**Keywords:** gastric cancer, microRNA, miR-208a-3p, PDCD4, apoptosis

## Abstract

Programmed cell death 4 (PDCD4) is a novel tumor suppressor gene and a promising target for anticancer therapies. PDCD4 is frequently downregulated in various human cancers; however, the molecular mechanism accounting for the loss expression of PDCD4 in cancers is not fully understood. In this study, we identified specific targeting sites for miR-208a-3p in the 3′-untranslated region (3′-UTR) of the PDCD4 gene which regulated PDCD4 expression. We demonstrated that miR-208a-3p suppressed apoptosis in gastric cancer cells by targeting PDCD4. We also showed that miR-208a-3p promoted the development of tumor growth in xenograft mice by negatively regulating PDCD4. Taken together, this study revealed a critical role for miR-208a-3p as an oncogenic miRNA in gastric carcinogenesis and it may provide a potential novel target for gastric cancer diagnosis and therapy.

## INTRODUCTION

Gastric cancer is one of the most frequently diagnosed cancers and the second leading cause of cancer-related death worldwide. An estimated 951,600 new gastric cancer cases and 723,100 deaths occurred in 2012 [1]. China presents the highest incidence of gastric cancer and highest rates of gastric cancer mortality [2]. Despite several strategies have been proposed for gastric cancer screening, most patients were diagnosed at advanced stage with dismal outcome [3]. More than half of all those diagnosed with locally advanced gastric cancer will experience recurrence [4]. Although several molecularly targeted drugs (e.g., trastuzumab for patients with HER-2-positive advanced gastric cancer) have been developed, most advanced gastric cancers have a poor prognosis, and new appropriate site for targeted therapy needs to be found.

Programmed cell death 4 (PDCD4) is a novel tumor suppressor gene that has been known as a promising target for future anticancer therapies for several years. The PDCD4 expression levels were shown to decrease in a number of different human tumors, including glioblastoma [5] and cancers of the stomach, pancreas, colon, lung, prostate, ovary and liver [6–10]. However, the molecular mechanism accounting for the loss expression of PDCD4 in human cancers is not fully understood. Interestingly, it has been reported that cumulative survival rate of gastric cancer patients with PDCD4 expression was significantly higher compared to the patients without PDCD4 expression [11], impling PDCD4 as a promising target for future anticancer therapies in gastric cancer.

microRNAs (miRNAs) are a family of small, non-coding RNAs that play an important role in the regulation of gene expression at the post-transcriptional level [12–14]. miRNAs bind to complementary sequences in the 3′-untranslated regions (3′-UTRs) of target mRNAs to induce degradation or translational repression of the target genes. Recent evidence has demonstrated that miRNAs can function as oncogenes or tumor suppressors via targeting cancer-related genes, and abnormal expression of miRNAs is a hallmark of human cancers [15]. Interestingly, some miRNAs that were previously thought to be tissue-specific and tumor-unrelated have also been linked to cancers currently. One such example is miR-208. miR-208 is widely known to play a critical role in the formation of cardiovascular and muscular diseases [16–18]. However, miR-208 has also been reported to be involved in human cancers occasionally, including pancreatic cancer [19], esophageal squamous cell carcinoma [20], hepatocellular carcinoma [21] and prostate carcinoma [22]. But in gastric cancer, the expression profile of miR-208a-3p has not been systematically investigated and the precise function of this miRNA in gastric tumorigenesis remains to be elucidated.

In this study, we identified PDCD4 as a direct target gene of miR-208a-3p and showed that miR-208a-3p inhibited PDCD4 expression directly. Consequently, miR-208a-3p suppressed the apoptosis of gastric cancer cells *in vitro* and accelerated gastric tumor growth *in vivo*.

## RESULTS

### Downregulation of PDCD4 protein in human gastric cancer tissues

**Figure 1:**
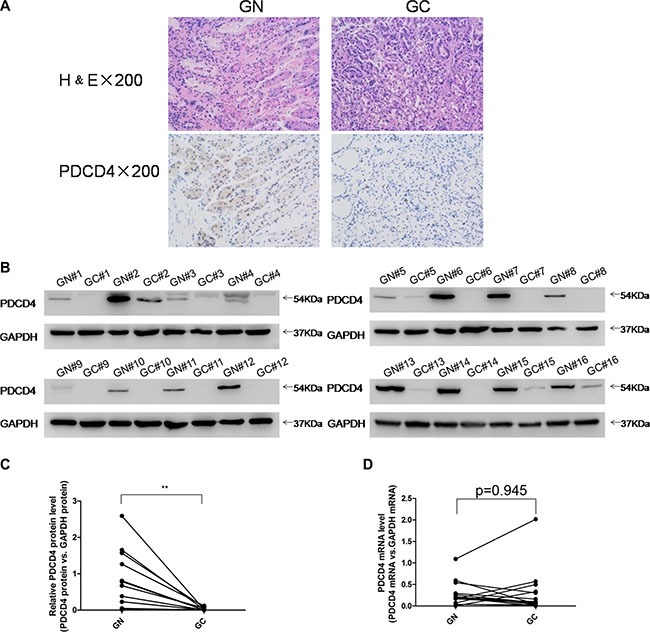
Expression patterns of PDCD4 in human gastric cancer tissues (**A**) H&E-stained sections and immunohistochemical staining for PDCD4 in gastric cancer tissue (GC) and gastric noncancerous tissue (GN) samples. (**B** and **C**) Western blotting analysis of PDCD4 protein levels in 16 pairs of GC and gastric GN samples. (A) representative image; (B) quantitative analysis. (**D**) Quantitative RT-PCR analysis of mRNA levels in 16 pairs of GC and GN samples (*P < 0.05; **P < 0.01).

To investigate the clinical relevance of PDCD4 in gastric cancer, we first examined PDCD4 expression in human gastric cancer tissues. Distinctly lower expression levels of PDCD4 were observed in gastric cancer tissues compared to matched adjacent normal tissues by immunohistochemistry (Figure 1A). Likewise, after measuring the PDCD4 protein levels in 16 pairs of gastric cancer tissues and adjacent noncancerous tissues by western blotting, the PDCD4 protein levels were consistently shown to be lower in the gastric cancer tissues (Figure 1B–1C and Supplementary Figure S1A). However, the PDCD4 mRNA levels did not differ significantly between the cancer and noncancerous tissues (Figure 1D and Supplementary Figure S1B). This disparity between PDCD4 protein and mRNA expression in gastric cancer tissues strongly suggests that a post-transcriptional mechanism is involved in PDCD4 regulation.

### Identification of conserved miR-208a-3p target sites within the 3′-UTR of PDCD4

**Figure 2:**
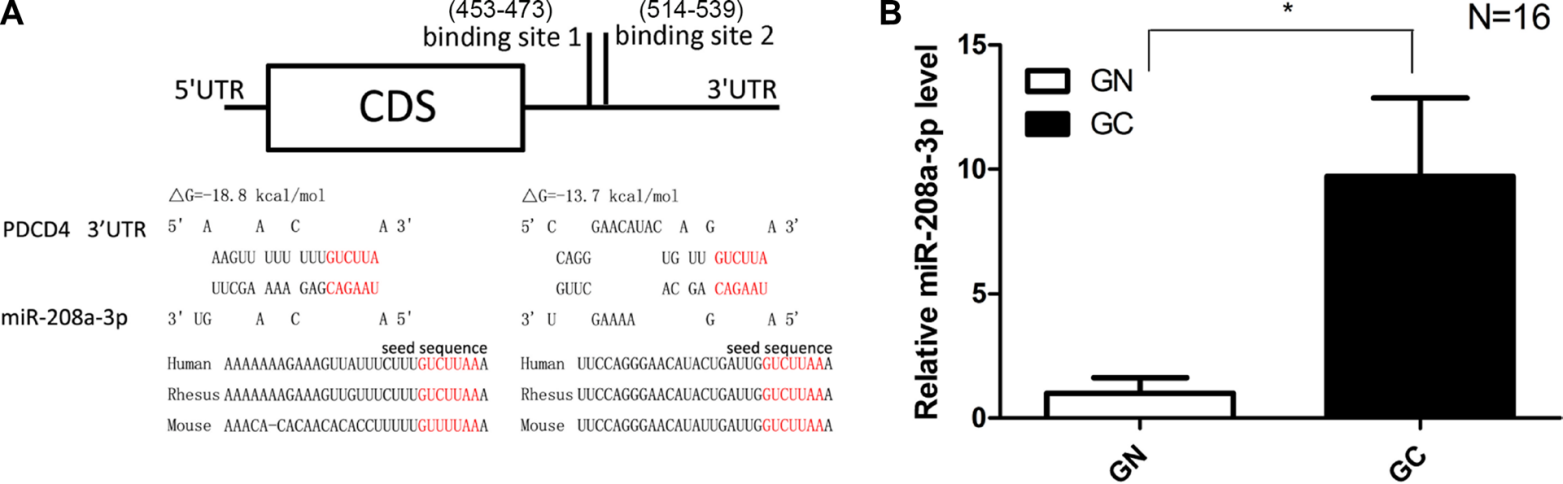
Prediction of the miR-208a-3p binding site within the PDCD4 3′-UTR (**A**) Schematic description of the hypothetical duplexes formed by the interactions between the binding sites in the PDCD4 3′-UTR (top) and miR-208a-3p (bottom). The predicted free energy value of each hybrid is indicated. The seed recognition sites are indicated in red, and all nucleotides in these regions are highly conserved in several species. (**B**) Quantitative RT-PCR analysis of the expression levels of miR-208a-3p in the same 16 pairs of GC and GN samples (*P < 0.05).

Because miRNAs play important roles in post-transcriptional regulation, it is quite likely that miRNAs regulate PDCD4 expression in human gastric cancer. Three computational algorithms (TargetScan [23], miRanda [24] and PicTar [25]) were used in combination to identify potential miRNAs that targeted PDCD4. Among the candidate miRNAs, miR-208a-3p was predicted to be a PDCD4 regulator by all three algorithms and was selected for further experimental verification. The predicted interaction between miR-208a-3p and the targeting sites within the PDCD4 3′-UTR is illustrated in Figure 2A. As shown in this figure, the 3′-UTR of PDCD4 contained two conserved binding sites for miR-208a-3p. The minimum free energy values of the hybrids were −18.8 and −13.7 kcal/mol, which were well within the range of genuine miRNA-target pairs. Moreover, there was perfect base-pairing between the seed region (the core sequence that encompasses the first 2–8 bases of the mature miRNA) and the cognate targets.

### Detection of an inverse correlation between miR-208a-3p and PDCD4 levels in gastric cancer tissues

Because miRNAs are generally thought to have expression patterns that are opposite to that of their targets [12, 13], we investigated whether miR-208a-3p expression was inversely correlated with PDCD4 expression in gastric cancer. After measuring the expression levels of miR-208a-3p in the same 16 pairs of gastric cancer tissues and matched noncancerous tissues, we identified that the miR-208a-3p levels were remarkably higher in the cancer tissues (Figure 2B). Thus, PDCD4 was regarded as a miR-208a-3p target based on both computational predictions and the inverse correlation between miR-208a-3p levels and PDCD4 protein levels in human gastric cancer.

### Validation of PDCD4 as a direct target of miR-208a-3p

**Figure 3:**
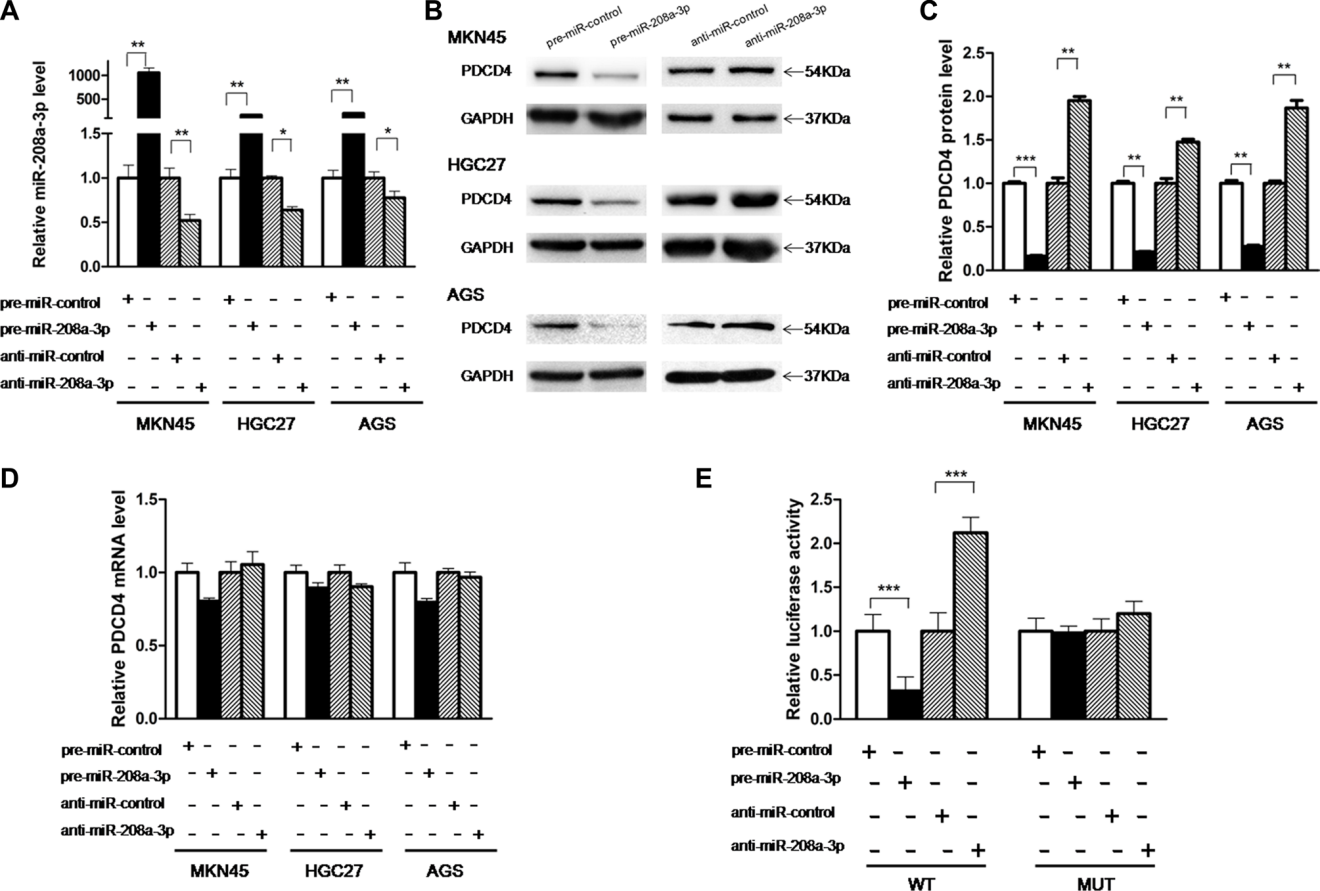
PDCD4 is a direct target of miR-208a-3p (**A**) Quantitative RT-PCR analysis of the expression levels of miR-208a-3p in MKN45, HGC-27 and AGS cells transfected with equal doses of the pre-miR-208a-3p, anti-miR-208a-3p or scrambled negative control RNAs (pre-miR-control or anti-miR-control). (**B** and **C**) Western blotting analysis of PDCD4 protein levels in MKN45, HGC-27 and AGS cells transfected with equal doses of the pre-miR-208a-3p, anti-miR-208a-3p or scrambled negative control RNAs. B: representative image; C: quantitative analysis. (**D**) Quantitative RT-PCR analysis of PDCD4 mRNA levels in MKN45, HGC-27 and AGS cells transfected with equal doses of the pre-miR-208a-3p, anti-miR-208a-3p or scrambled negative control RNAs. (**E**) Direct recognition of the PDCD4 3′-UTR by miR-208a-3p. Firefly luciferase reporters containing either wild-type (WT) or mutant (MUT) miR-208a-3p binding sites in the PDCD4 3′-UTR were co-transfected into AGS cells with equal doses of the pre-miR-208a-3p, anti-miR-208a-3p or scrambled negative control RNAs. The cells were assayed using a luciferase assay kit 24 h post-transfection. Firefly luciferase values were normalized to β-galactosidase activity, and the results were calculated as the ratio of firefly luciferase activity in the miR-208a-3p-transfected cells normalized to the control RNA-transfected cells (*p < 0.05; **p < 0.01; ***p < 0.005).

The correlation between miR-208a-3p and PDCD4 was examined by evaluating PDCD4 expression in human gastric cancer cell lines (MKN45, HGC-27 and AGS) after overexpression or knockdown of miR-208a-3p. Cellular miR-208a-3p levels were significantly increased in MKN45, HGC-27 and AGS cells when these cells were transfected with pre-miR-208a-3p, and miR-208a-3p levels were decreased when these cells were transfected with anti-miR-208a-3p (Figure 3A). Consequently, the expression of the PDCD4 protein was distinctly reduced by the overexpression of miR-208a-3p and increased by the knockdown of miR-208a-3p in MKN45, HGC-27 and AGS cells (Figure 3B and 3C). To clarify the regulatory level at which miR-208a-3p influenced PDCD4 expression, we repeated the above experiments and examined the expression of PDCD4 mRNA after transfection. Overexpression or knockdown of miR-208a-3p did not affect PDCD4 mRNA levels in the three cell lines (Figure 3D). These results demonstrated that miR-208a-3p specifically regulate PDCD4 expression at the post-transcriptional level.

To investigate whether miR-208a-3p regulates PDCD4 expression through binding to the 3′-UTR of PDCD4 mRNA, the entire 3′-UTR of PDCD4 mRNA containing the presumed miR-208a-3p binding sites was fused downstream of the firefly luciferase gene in a reporter plasmid. The resulting plasmid was transfected into AGS cells along with pre-miR-208a-3p, anti-miR-208a-3p or scrambled negative control RNAs. As expected, luciferase reporter activity was reduced to 40% in cells transfected with pre-miR-208a-3p, whereas inhibition of miR-208a-3p resulted in a two-fold increase in reporter activity (Figure 3E). Next, we engineered a mutant plasmid by introducing point mutations into the corresponding complementary seed site in the PDCD4 3′-UTR to eliminate the predicted miR-208a-3p binding sites. Luciferase activity of the mutated luciferase reporter was unaffected by either the overexpression or knockdown of miR-208a-3p (Figure 3E), suggesting that the binding sites strongly contribute to the miRNA:mRNA interaction. Taken together, our results indicate that miR-208a-3p directly recognizes and binds to the 3′-UTR of the PDCD4 transcript and inhibits PDCD4 translation.

### miR-208a-3p suppresses apoptosis of gastric cancer cells by targeting PDCD4

**Figure 4:**
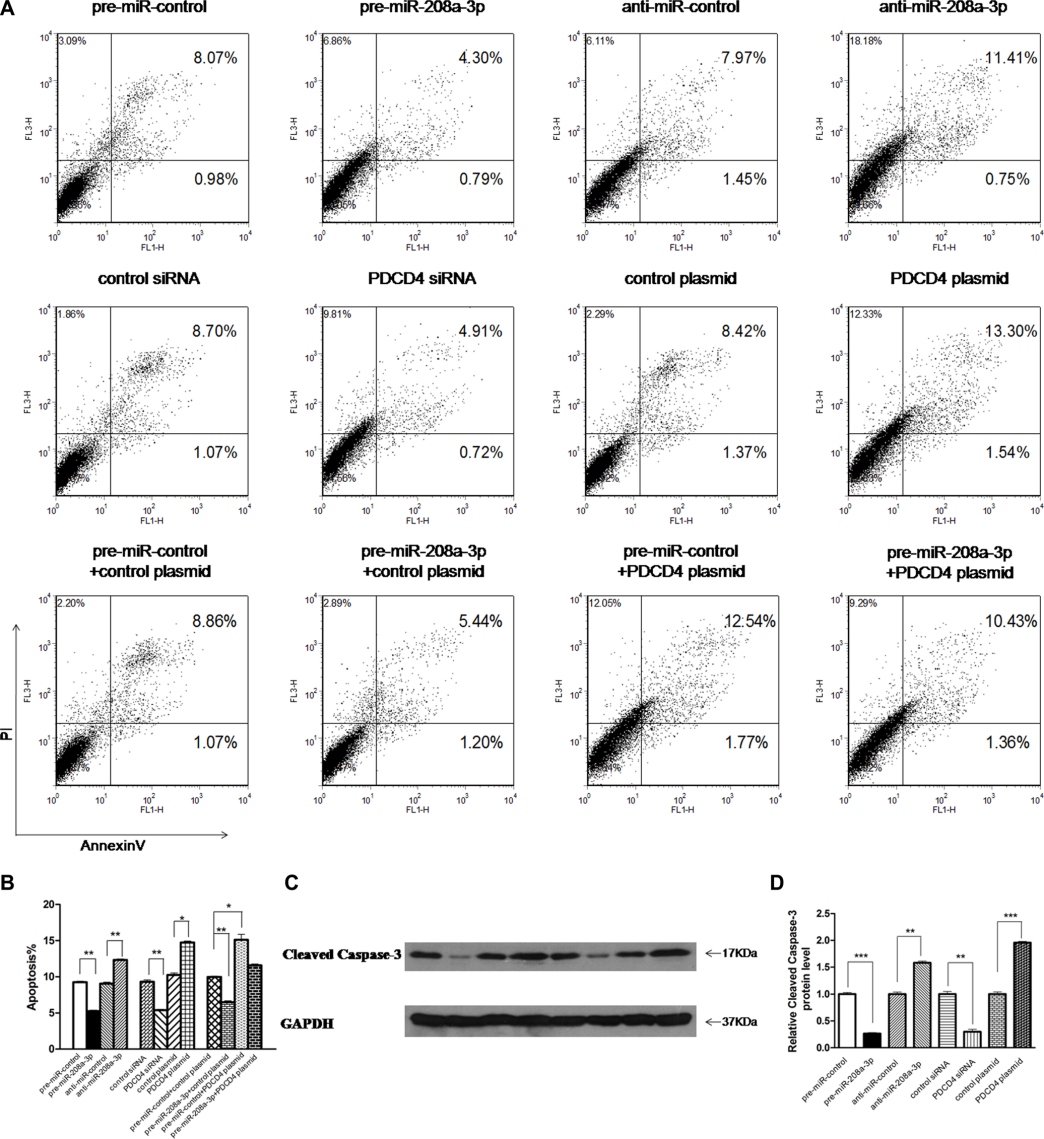
Effect of miR-208a-3p and PDCD4 on the apoptosis of gastric cancer cells (**A**) The apoptosis assay was performed 24 h after the transfection of MKN45 cells with equal doses of the pre-miR-208a-3p, anti-miR-208a-3p or scrambled negative control RNAs (upper panel), or with equal doses of control siRNA, PDCD4 siRNA, control plasmid or PDCD4 overexpression plasmid (middle panel), or with equal doses of pre-miR-control plus control plasmid, pre-miR-control plus PDCD4 overexpression plasmid, pre-miR-208a-3p plus control plasmid, or pre-miR-208a-3p plus PDCD4 overexpression plasmid (lower panel). (**B**) Quantitative analysis of the apoptotic cell ratio in panel A. (**C** and **D**) Western blotting analysis of Cleaved Caspase-3 protein levels in MKN45 cells transfected with equal doses of the pre-miR-208a-3p, anti-miR-208a-3p or scrambled negative control RNAs, or with equal doses of control siRNA, PDCD4 siRNA, control plasmid or PDCD4 overexpression plasmid. (C) representative image; (D) quantitative analysis. (*p < 0.05; **p < 0.01; ***p < 0.005).

Next, we focused on studying the role of miR-208a-3p in regulating PDCD4. PDCD4 was reported to intensively promote cell apoptosis, and play an important role in mediating the sensitivity of gastric cancer cells to TRAIL-induced apoptosis through FLIP suppression [26]. We investigated whether the overexpression or knockdown of miR-208a-3p or PDCD4 would affect cell apoptosis in MKN45 cells induced by TRAIL using flow cytometry analysis. To knock down PDCD4, a siRNA targeting PDCD4 was designed and transfected into MKN45 cells. To overexpress PDCD4, an expression plasmid designed to specifically express the full-length ORF of PDCD4 without the miR-208a-3p-responsive 3′-UTR was constructed and transfected into MKN45 cells. Efficient knockdown and overexpression of PDCD4 in MKN45 cells is shown in Supplementary Figure S2. In substantial consistence with the hypothesis that miR-208a-3p functions as an oncogenic miRNA, the apoptosis assay showed that the percentage of apoptotic cells was significantly lower in cells transfected with pre-miR-208a-3p but higher in cells transfected with anti-miR-208a-3p (Figure 4A and 4B). Similarly, MKN45 cells transfected with the PDCD4 siRNA showed suppression of cell apoptosis, whereas overexpression of PDCD4 significantly promoted cell apoptosis (Figure 4A and 4B). Thus, miR-208a-3p and PDCD4 exerted opposing effects on cell apoptosis. When MKN45 cells were simultaneously transfected with pre-miR-208a-3p and the PDCD4 overexpression plasmid, PDCD4 dramatically rescued the suppressive effect of miR-208a-3p on cell apoptosis (Figure 4A and 4B). Additionally, the apoptosis assay showed the similar results when MKN45 cells treated with RPMI 1640 medium without FBS to induce apoptosis after transfection (Supplementary Figure S3). Moreover, we also investigated the activation of caspase-3 through western blotting. As shown in Figure 4C and 4D, the activation of caspase-3 significantly decreased in cells transfected with pre-miR-208a-3p and the PDCD4 siRNA, but increased in cells transfected with anti-miR-208a-3p and the PDCD4 overexpression plasmid (Figure 4C and 4D). These results reveal that PDCD4 is crucial for the apoptosis of gastric cancer cells and that miR-208a-3p is able to suppress cell apoptosis by silencing PDCD4.

### The influence of miR-208a-3p and PDCD4 on the growth of gastric cancer cells *in vivo*

**Figure 5:**
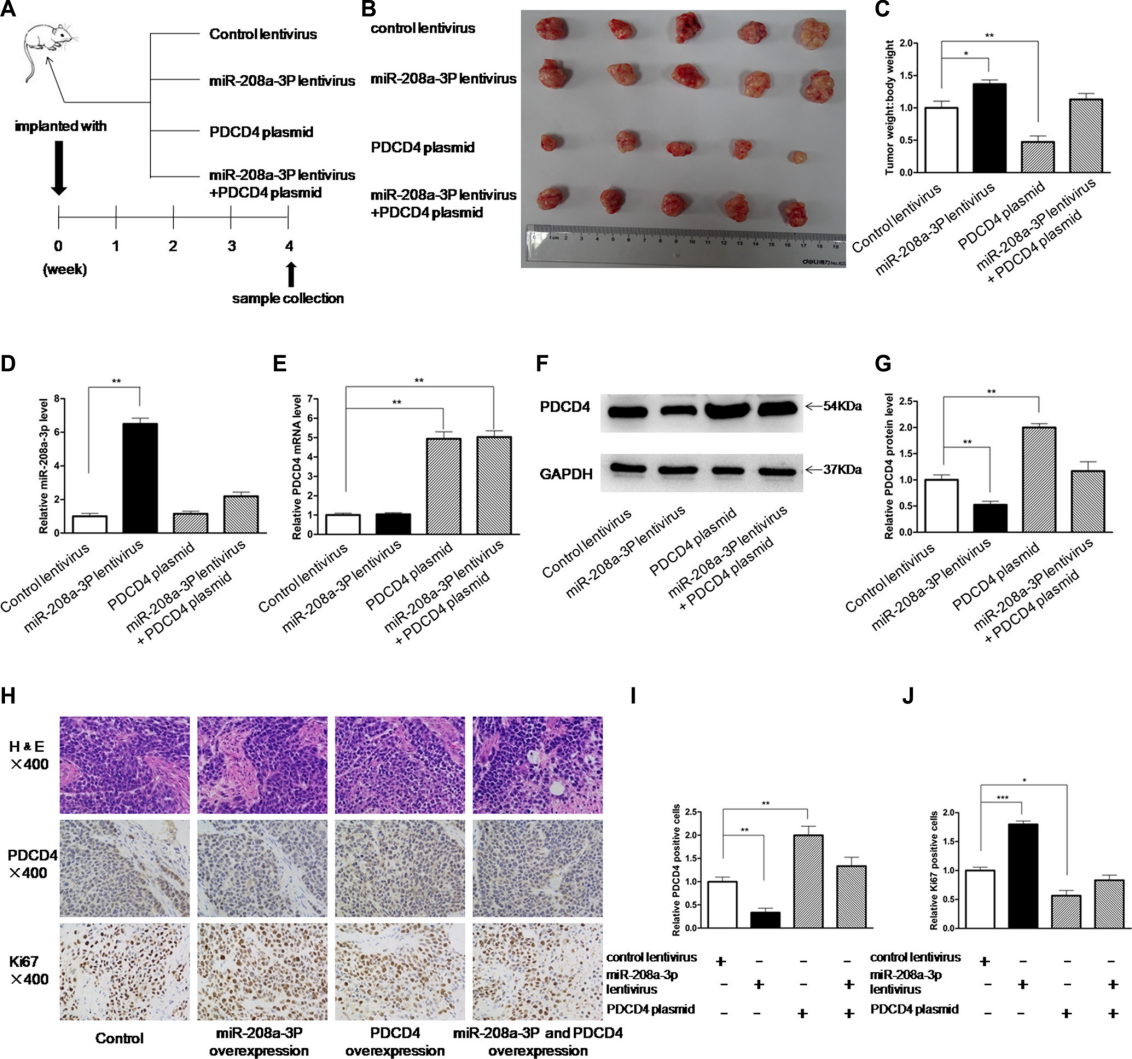
Effects of miR-208a-3p and PDCD4 on the growth of gastric cancer cell xenografts in mice (**A**) Flow chart of the experimental procedure. MKN45 cells were infected with a control lentivirus or a miR-208a-3p overexpression lentivirus, or transfected with a PDCD4 overexpression plasmid, or co-transfected with a miR-208a-3p overexpression lentivirus and a PDCD4 overexpression plasmid. MKN45 cells (2 × 10^6^ cells per 0.1 mL) with different treatments were implanted subcutaneously into 6-week-old SCID mice (5 mice per group), and the tumor growth was evaluated on day 28 after cell implantation. (**B**) Representative images of the tumors from the implanted mice. (**C**) Quantitative analysis of the tumor weights. (**D**) Quantitative RT-PCR analysis of miR-208a-3p levels in the tumors from implanted mice. (**E**) Quantitative RT-PCR analysis of PDCD4 mRNA levels in the tumors from implanted mice. (**F** and **G**) Western blotting analysis of PDCD4 protein levels in the tumors from implanted mice. (F) representative image; (G) quantitative analysis. (**H**–**J**) H&E-stained sections and immunohistochemical staining for Ki-67 and PDCD4 in the tumors from implanted mice. (H) representative image; (I) and (J) quantitative analysis (*p < 0.05; **p < 0.01; ***p < 0.005).

Subsequently, we evaluated the biological effects of miR-208a-3p and PDCD4 on the growth of gastric cancer cells in a gastric cancer xenograft mouse model. We first generated a viral expression construct to express miR-208a-3p. A 300-bp fragment containing the genomic miR-208a-3p sequence was cloned into a lentiviral expression vector, and MKN45 cells were infected with the lentivirus to overexpress miR-208a-3p. The expression of mature miR-208a-3p was found to be 4-fold higher than the endogenous miRNA levels (Supplementary Figure S4). Next, MKN45 cells were infected with a control lentivirus or the miR-208a-3p overexpression lentivirus, or transfected with the PDCD4 overexpression plasmid, or co-transfected with the miR-208a-3p overexpression lentivirus and PDCD4 overexpression plasmid, and then the infected or transfected cells were subcutaneously injected into SCID mice. After four weeks of xenograft growth *in vivo*, the mice were sacrificed and the weight of the tumors was measured (Figure 5A). We observed a significant increase in the size and weight of the tumors in the miR-208a-3p-overexpressing group compared to the control group, whereas the size and weight of the tumors in the group implanted with the PDCD4-overexpression plasmid were dramatically decreased (Figure 5B and 5C). Additionally, PDCD4 overexpression attenuated the promotive effect of miR-208a-3p on tumor growth (Figure 5B and 5C), suggesting that miR-208a-3p might promote tumor growth by silencing PDCD4. We also extracted total RNA and protein from each xenograft and analyzed miR-208a-3p and PDCD4 expression. Tumors from the miR-208a-3p-overexpression group showed a significant increase in the miR-208a-3p levels compared to tumors from the control group (Figure 5D). Likewise, PDCD4 mRNA levels were increased in the tumors from the PDCD4-overexpressing group (Figure 5E). Tumors from the miR-208a-3p-overexpressing group displayed reduced PDCD4 protein levels compared to tumors from the control group, whereas the PDCD4-overexpressing group showed elevated PDCD4 protein levels (Figure 5F and 5G). Tumors with both miR-208a-3p and PDCD4 overexpression exhibited significantly higher levels of PDCD4 compared to tumors with miR-208a-3p overexpression (Figures 5F and 5G), suggesting that PDCD4 overexpression could rescue the PDCD4 suppression caused by miR-208a-3p. Furthermore, Hematoxylin and eosin (H&E) staining of xenograft tissues showed more cell pathological mitosis in the group implanted with the miR-208a-3p lentivirus compared with the control group, whereas confluent necrotic areas and more apoptotic cells were observed in the xenografts from the PDCD4-overexpressing group (Figure 5H). Xenografts with both miR-208a-3p and PDCD4 overexpression exhibited promoted cell apoptosis compared to xenografts with miR-208a-3p overexpression (Figure 5H), suggesting that PDCD4 overexpression could attenuate the suppressive effect of miR-208a-3p on cell apoptosis. Immunohistochemical staining also revealed the presence of lower levels of PDCD4 in the tumors from mice implanted with miR-208a-3p-overexpressing cells, whereas the tumors from the PDCD4-overexpressing mice showed increased PDCD4 protein levels (Figure 5H and 5I). Finally, the proliferative activity of tumor cells was assessed by immunocytochemistry with the mouse monoclonal antibody Ki-67. The cell proliferation rate was decreased in the group implanted with the PDCD4 plasmid and increased in the group implanted with the miR-208a-3p lentivirus (Figure 5H and 5J). Similarly, PDCD4 overexpression attenuated the promotive effect of tumor growth caused by miR-208a-3p overexpression (Figure 5H and 5J). These results were consistent with the findings of the *in vitro* assays, which firmly validated the oncomiR role of miR-208a-3p in tumorigenesis through the targeting of PDCD4.

## DISCUSSION

Gastric cancer is the second most common cause of death from cancer worldwide [27]. Despite the enormous advances in treatments, most patients with advanced gastric cancer exhibit a poor prognosis. The main treatment of gastric cancer is surgery in combination with chemotherapy and/or radiotherapy. Molecular and gene profiling is the key to defining subsets of patients in the future [4]. The addition of trastuzumab to a cisplatin and fluoropyrimidine chemotherapy doublet is a valid first-line treatment option for HER-2-positive advanced gastric cancer [28]. However, the current methods of molecular targeted therapy are extremely limited.

PDCD4 is a novel tumor suppressor gene, and its protein product plays a role in suppression of tumorigenesis and tumor invasion. PDCD4 is thought to be an attractive candidate for future antitumor therapies. Lost expression of PDCD4 protein has been identified in many different human cancers, such as cancers of stomach, pancreas, colon, lung, prostate, ovary and liver [6–10]. Low expression of PDCD4 is also associated with poor prognosis [11]. Restoring PDCD4 production in tumor cells can be used as a method to control oncologic disease [29]. Previous studies indicate that PDCD4 promotes cell apoptosis. PDCD4 is able to suppress expression of FLICE-inhibiting protein (FLIP), a negative regulator of apoptosis [26]. PDCD4 expression has been suggested to be increased during apoptosis in response to different inducers [30]. However, how PDCD4 is regulated during tumorigenesis is still unclear. Recently, Motoyama *et al.* and Cao *et al.* revealed PDCD4 is repressed by miR-21 in gastric cancer [31, 32]. In this study, we showed that silencing PDCD4 expression using siRNA could suppress cell apoptosis in gastric cancer cells, whereas overexpressing PDCD4 produced an opposite effect. It seems that PDCD4 functions as an antioncogenic protein during tumorigenesis. Simultaneously, we showed that PDCD4 protein was frequently downregulated in gastric cancer tissues, and we identified discordance between the PDCD4 protein and mRNA levels in human gastric cancer tissues. The results suggest that a post-transcriptional regulation mechanism is involved in PDCD4 repression. One of the most important modes of post-transcriptional regulation is the repression of mRNA transcripts by miRNAs. Therefore, we searched for miRNAs that could target PDCD4 and experimentally validated PDCD4 as a target of miR-208a-3p. Additionally, we also found miR-21 levels were remarkably higher in the cancer tissues like the previous study [31, 32] (Supplementary Figure S5). Therefore, modulation of PDCD4 by miR-208a-3p and miR-21 might explain, at least in part, why the upregulation of miR-208a-3p and miR-21 during tumorigenesis can silence PDCD4 and promote tumor cell growth and gastric cancer formation.

Abnormal expression of miRNAs has been detected in a number of tumor types, and miRNAs are reported to be associated with human carcinogenesis and cancer progression. Thus, miRNAs are regarded as direct therapeutic targets for cancers, and understanding the molecular and cellular pathways controlling miRNA biogenesis and how these mechanisms go awry in cancer will identify promising therapeutic targets [33]. The previous studies indicate that miR-208-3p is dysregulated in some cardiovascular and muscular diseases [16–18]. However, there are few studies exploring the expression and function of miR-208-3p in cancers, except some occasional reports in pancreatic cancer [19], esophageal squamous cell carcinoma [20], hepatocellular carcinoma [21] and prostate carcinoma [22]. In agreement with our hypothesis, miR-208-3p has also been shown to be upregulated and behave as an oncogenic miRNA in these human tumor types. In this study, we detected an inverse correlation between miR-208a-3p levels and PDCD4 protein levels in human gastric cancer tissues and paired noncancerous tissues. By knocking down or overexpressing miR-208a-3p in gastric cancer cells, we validated that miR-208a-3p directly inhibited PDCD4 translation. In addition, we showed that the cellular phenotypes especially cell apoptosis was regulated by miR-208a-3p via negatively regulating PDCD4. The results revealed that miR-208a-3p inhibited PDCD4 expression and consequently suppress cell apoptosis, both *in vitro* and *in vivo*. The results generalized a novel regulatory network employing miR-208a-3p and PDCD4 to fine-tune cell apoptosis. We also provided evidence that restoration of PDCD4 expression can reverse miR-208a-3p-suppressed cell apoptosis, suggesting that the targeting of PDCD4 is one mechanism by which the miR-208a-3p exerts its tumorigenesis function.

In summary, this study not only reveals a critical role for miR-208a-3p as an oncogenic miRNA in gastric carcinogenesis but also explores the molecular mechanisms by which miR-208a-3p contributes to gastric cancer progression. Regulation of PDCD4 by miR-208a-3p may explain why the upregulation of miR-208a-3p during carcinogenesis can promote cancer progression. This study also provides a potential novel target for gastric cancer therapy.

Future research emphasis is needed to characterize the feasibility of targeting miR-208-3p in gastric cancer therapy and developing simplified and cost-effective manipulation methods.

## MATERIALS AND METHODS

### Cells and human tissues

The human gastric cancer cell lines MKN45, HGC-27 and AGS were purchased from the Shanghai Institute of Cell Biology, Chinese Academy of Sciences (Shanghai, China). The cells were cultured in RPMI 1640 medium supplemented with 10% fetal bovine serum (FBS, Gibco, Carlsbad, CA, USA) in a 5% CO_2_, water-saturated atmosphere. The 16 paired samples of human gastric cancer and their matched adjacent noncancerous tissues were derived from patients undergoing a surgical procedure at the Nanjing Drum Tower Hospital Clinical College of Nanjing Medical University (Nanjing, China). All of the patients provided written consent, and the Ethics Committee from Nanjing Medical University approved all aspects of this study. Tissue fragments were immediately frozen in liquid nitrogen at the time of surgery and stored at −80°C. The clinical features of the patients are listed in Supplementary Table S1.

### RNA isolation and quantitative RT-PCR

Total RNA was extracted from cultured cells and human tissues using the TRIzol Reagent (Invitrogen, Carlsbad, CA) according to the manufacturer's instructions. Assays to quantify miRNAs were performed using Taqman miRNA probes (Applied Biosystems, Foster City, CA, USA) according to the manufacturer's instructions. Briefly, 1 μg of total RNA was reverse-transcribed into cDNA using the AMV reverse transcriptase (TaKaRa, Dalian, China) and a stem-loop RT primer (Applied Biosystems). The reaction conditions were as follows: 16°C for 30 min, 42°C for 30 min and 85°C for 5 min. Real-time PCR was performed using a Taqman PCR kit on an Applied Biosystems 7300 Sequence Detection System (Applied Biosystems). The reactions were incubated in a 96-well optical plate at 95°C for 10 min, followed by 40 cycles of 95°C for 15 s and 60°C for 1 min. All of the reactions were run in triplicate. After the reaction, the cycle threshold (C_T_) data were determined using fixed threshold settings, and the mean C_T_ was determined from the triplicate PCRs. A comparative C_T_ method was used to compare each condition to the controls. The relative levels of miRNAs in the cells and tissues were normalized to U6 small nuclear RNA. The amount of miRNA relative to the internal control U6 was calculated with the equation 2^−ΔΔCT^, in which ΔΔC_T_ = (C_T miR-208_ - C_T U6_)_tumor_ - (C_T miR-208_ - C_T U6_)_control_. To quantify PDCD4 mRNA expression, 1 μg of total RNA was reverse-transcribed into cDNA using oligo dT and the AMV reverse transcriptase (TaKaRa, Dalian, China) with the following conditions: 16°C for 30 min, 42°C for 30 min and 85°C for 5 min. Next, real-time PCR was performed with the RT product, SYBR Green Dye (Invitrogen) and specific primers for PDCD4 and GAPDH. The sequences of the primers were as follows: PDCD4 (sense): 5′-GTTGGCAGTATCCTTAGCATTGG-3′; PDCD4 (antisense): 5′-TCCACATCAGTTGTGCTCATTAC-3′; GAPDH (sense): 5′-CGAGCCACATCGCTCAGACA-3′; and GAPDH (antisense): 5′-GTGGTGAAGACGC CAGTGGA-3′. The reactions were incubated at 95°C for 5 min, followed by 40 cycles of 95°C for 30 s, 60°C for 30 s and 72°C for 1 min. After the reactions were completed, the C_T_ values were determined by setting a fixed threshold. The relative amount of PDCD4 mRNA was normalized to GAPDH using a method similar to that described above.

### Protein extraction and western blotting

Cells and tissues were lysed in RIPA Lysis buffer (Beyotime, Shanghai, China) supplemented with a Protease and Phosphatase Inhibitor Cocktail (Thermo Scientific 78440) on ice for 30 min and then centrifuged at 12,000 × g at 4°C for 10 min. The supernatant was collected, and the protein concentration was calculated with a Pierce BCA protein assay kit (Thermo Scientific, Rockford, IL, USA). Proteins were separated by SDS-PAGE (Bio-Rad). After electrophoresis, the proteins were electrotransferred to PVDF membranes (Bio-Rad) and then blocked in phosphate-buffered saline/Tween-20 containing 5% non-fat milk for 1 h. The membranes were then incubated with primary antibodies at 4°C for 12 h. After four washes in TBST, the membranes were incubated with horseradish peroxidase-conjugated secondary antibody for 1 h at room temperature. After four washes, the membranes were incubated with the SuperSignal West Pico chemiluminescence substrate (Pierce). The PDCD4 protein levels were analyzed by western blotting with a monoclonal anti-human PDCD4 antibody (k4C1, sc-130545). The Cleaved Caspase-3 protein levels were analyzed by western blotting with a polyclonal anti-human Cleaved Caspase-3 antibody (#9661). The protein levels were normalized by probing the same blots with a GAPDH antibody (FL-335, sc-25778). The anti-PDCD4 and anti-GAPDH antibodies were purchased from Santa Cruz Biotechnology (CA, USA), and the anti-Cleaved Caspase-3 antibodies were purchased from Cell Signaling Technology (MA, USA).

### Overexpression or knockdown of miR-208a-3p

Overexpression of miR-208a-3p was achieved by transfecting gastric cancer cells with a miRNA mimic (a synthetic RNA oligonucleotide duplex mimicking the miRNA precursor). Knockdown of miR-208a-3p was achieved by transfecting a miRNA inhibitor (a chemically modified single-stranded antisense oligonucleotide designed to specifically target the mature miRNA). Synthetic mimic (pre-miR-208a-3p), inhibitor (anti-miR-208a-3p) and scrambled negative control RNA (pre-miR-control and anti-miR-control) were purchased from RiboBio (Guangzhou, China). MKN45, HGC-27 and AGS cells were seeded in 6-well plates or 60 mm dishes using RPMI 1640 media supplemented with 10% FBS. The cells were transfected with Lipofectamine 3000 (Invitrogen) using Opti-MEM Reduced Serum Medium (Gibco, Carlsbad, CA, USA) on the following day when the cells were approximately 70% confluent. For miRNA overexpression, equal amounts of pre-miR-208a-3p or pre-miR-control were used in each well. For miRNA knockdown, equal amounts of anti-miR-208a-3p or anti-miR-control were used. After 6 h, the media was changed to RPMI 1640 supplemented with 2% FBS. The cells were harvested 24 h or 48 h after the transfection for the isolation of total RNA and protein, respectively.

### Luciferase reporter assay

A sequence containing the presumed miR-208a-3p binding site was designed from the human PDCD4 3′-untranslated region (3′-UTR). The sequence was inserted into the p-MIR-reporter plasmid (Ambion). The insertion was confirmed to be correct by sequencing. To test the binding specificity, the sequences that interacted with the seed sequence of miR-208a-3p were mutated (from GUCUUAA to CAGAAUU), and the mutant PDCD4 3′-UTR was inserted into an equivalent luciferase reporter. For the luciferase reporter assays, AGS cells were cultured in 24-well plates, and each well was transfected with 0.4 μg of firefly luciferase reporter plasmid, 0.4 μg of a β-galactosidase (β-gal) expression plasmid (Ambion), and equal amounts (20 pmol) of pre-miR-208a-3p, anti-miR-208a-3p, or the scrambled negative control RNAs using Lipofectamine 2000 (Invitrogen). The β-gal plasmid was used as a transfection control. The cells were assayed using a luciferase assay kit 24 h post-transfection (Promega, Madison, WI, USA).

### Plasmid construction and siRNA interference assay

A mammalian expression plasmid encoding the full-length human PDCD4 open reading frame without the miR-208a-3p-responsive 3′-UTR was purchased from Invitrogen. An empty plasmid served as the negative control. The siRNA (sequence: GCGGAAAUGUUAAGAGAUU) targeting human PDCD4 cDNA was designed and synthesized by GenePharma (Shanghai, China). A scrambled siRNA was included as a negative control. Overexpression plasmid or siRNA were transfected into MKN45 cells using Lipofectamine 3000 (Invitrogen) according to the manufacturer's instructions. Total RNA and protein were isolated 24 h or 48 h after transfection. The PDCD4 mRNA and protein expression levels were assessed by quantitative RT-PCR and Western blotting, respectively.

### Cell apoptosis assay

Twenty-four hours after transfection with pre-miR-208a-3p, anti-miR-208a-3p, PDCD4 siRNA and the overexpression plasmid, MKN45 cells were treated with RPMI 1640 medium with 2% FBS containing TRAIL(20ng/ml) for 24 h to induce apoptosis. The cells were washed twice with cold PBS and resuspended in 1 × binding buffer at a concentration of 1 × 10^6^ cells/mL according to the instructions of the FITC-Annexin V Apoptosis Detection Kit I (BD Biosciences). The cells (1 × 10^5^ cells) were transferred to a 5 mL culture tube, and FITC-Annexin V and propidium iodide (PI) were added. The cells were incubated for 15 min at room temperature in the dark and analyzed by flow cytometry (BD Biosciences) within 1 h of staining. Tumor necrosis factor-related apoptosis induced ligand (TRAIL) were purchased from peprotech (NJ, USA).

### Tumor xenografts in mice

All animal care and handling procedures were performed in accordance with the National Institutes of Health's Guide for the Care and Use of Laboratory Animals and were approved by the Institutional Review Board of Nanjing University (Nanjing, China). Six-week-old male SCID (severe combined immune deficiency) mice (*nu*/*nu*) were purchased from the Model Animal Research Center of Nanjing University (Nanjing, China) and maintained under specific pathogen-free conditions at Nanjing University. MKN45 cells were infected with a control lentivirus or a lentivirus to overexpress miR-208a-3p, or transfected with a PDCD4 overexpression plasmid, or co-transfected with a miR-208a-3p overexpression lentivirus and a PDCD4 overexpression plasmid. After infection and transfection, the cells were injected subcutaneously into mice (2 × 10^6^ cells per mouse, 5 mice per group). The mice were sacrificed after four weeks. After the tumors were separated from the animals, the weight of the tumors was measured. Parts of the tissues were used for protein and total RNA extraction, and the remainder were fixed in 4% paraformaldehyde for 24 h and then processed for Hematoxylin and eosin (H&E) staining and immunohistochemical staining for PDCD4 and Ki-67.

### Statistical analysis

All of the images of western blot and cell apoptosis were representatives of at least three independent experiments. Quantitative RT-PCR assay and luciferase reporter assay were performed in triplicate, and each experiment was repeated several times. The data shown below are the mean ± SD of at least three independent experiments. The differences were considered statistically significant at *p* < 0.05 using Student's *t*-test (two-tailed). All the analyses were performed with the SPSS software (version 19.0) (IBM Corporation, New York, NY, USA).

## SUPPLEMENTARY MATERIALS FIGURES AND TABLES


