# Supplementary material for: miR-208a-3p suppresses cell apoptosis by targeting PDCD4 in gastric cancer
Source: Oncotarget. 2016 Sep 13;7(41):67321–32. doi: 10.18632/oncotarget.12006 (PMC5341878; doi:10.18632/oncotarget.12006)
Supplement: Supplementary file 1 [file oncotarget-07-67321-s001.pdf]

miR-208a-3p suppresses cell apoptosis by targeting PDCD4 in gastric cancer

Supplementary Materials

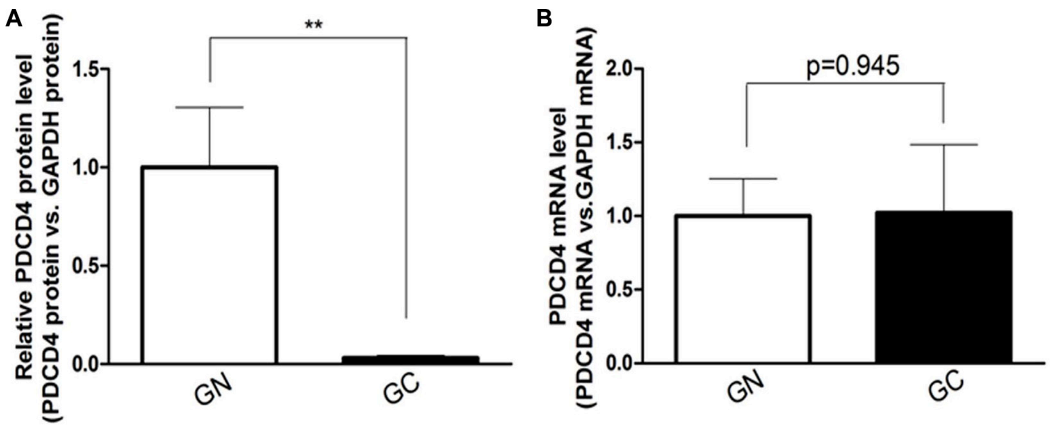

Supplementary Figure S1: Expression patterns of PDCD4 in human gastric cancer tissues (bar graphs of the average values). (**A**) Quantitative western blotting analysis of PDCD4 protein levels in 16 pairs of GC and gastric GN samples. (**B**) Quantitative RT-PCR analysis of mRNA levels in 16 pairs of GC and GN samples ($**P < 0.01$).

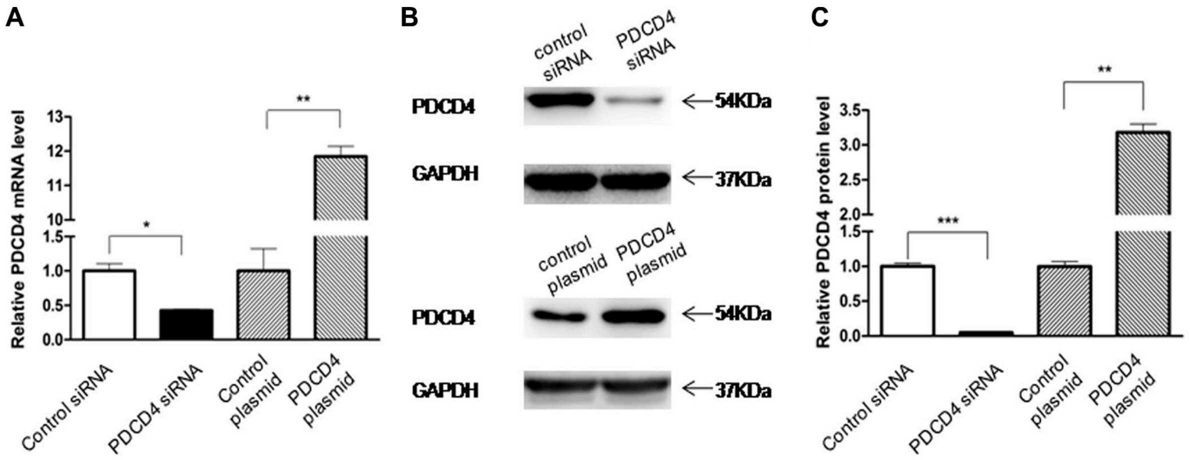

Supplementary Figure S2: Evaluation of the knockdown and overexpression efficiency of PDCD4 in MKN45 cells. (**A**) Quantitative RT-PCR analysis of PDCD4 mRNA levels in MKN45 cells transfected with PDCD4 siRNA or the scrambled negative control siRNA, or with the PDCD4 overexpression plasmid or control plasmid. (**B** and **C**) Western blotting analysis of the PDCD4 protein level in MKN45 cells transfected with PDCD4 siRNA or the scrambled negative control siRNA, or with the PDCD4 overexpression plasmid or control plasmid. (B) representative image; (C) quantitative analysis ($*p < 0.05$; $**p < 0.01$).

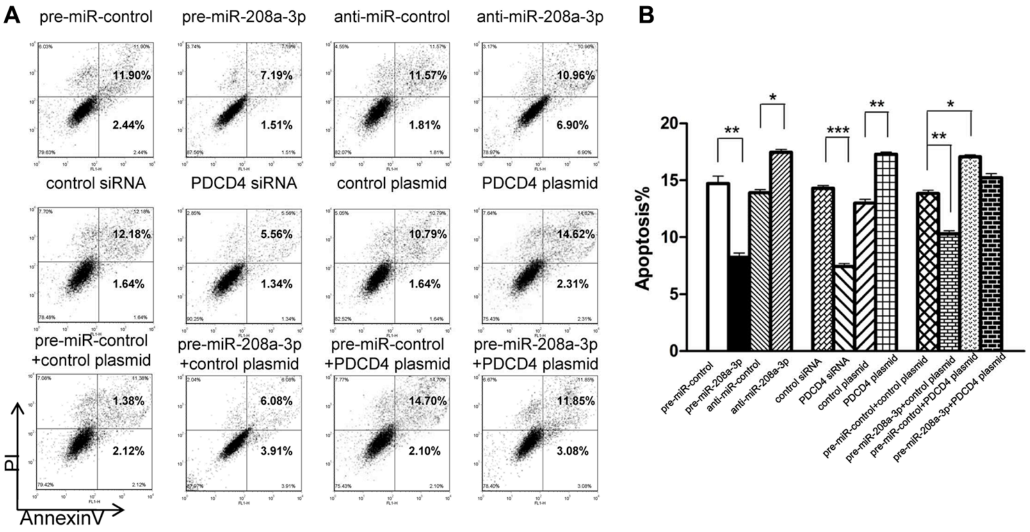

Supplementary Figure S3: Effect of miR-208a-3p and PDCD4 on the apoptosis of gastric cancer cells (RPMI 1640 medium without FBS for 24 h to induce apoptosis). (**A**) The apoptosis assay was performed 24 h after the transfection of MKN45 cells with equal doses of the pre-miR-208a-3p, anti-miR-208a-3p or scrambled negative control RNAs (upper panel), or with equal doses of control siRNA, PDCD4 siRNA, control plasmid or PDCD4 overexpression plasmid (middle panel), or with equal doses of pre-miR-control plus control plasmid, pre-miR-control plus PDCD4 overexpression plasmid, pre-miR-208a-3p plus control plasmid, or pre-miR-208a-3p plus PDCD4 overexpression plasmid (lower panel). (**B**) Quantitative analysis of the apoptotic cell ratio in panel A. (*$p < 0.05$; **$p < 0.01$; ***$p < 0.005$).

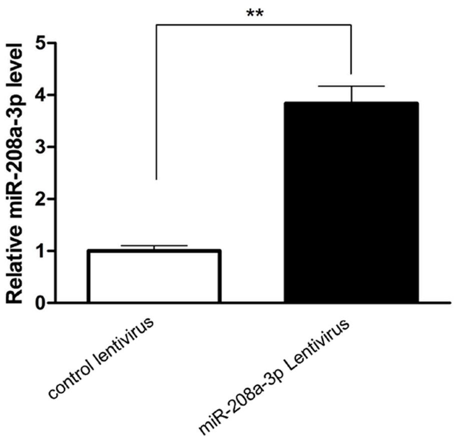

Supplementary Figure S4: The efficiency of miR-208a-3p lentiviral infection. Quantitative RT-PCR analysis was performed to measure the expression levels of miR-208a-3p in MKN45 cells after infection with a control lentivirus or a lentivirus to overexpress miR-208a-3p (**$p < 0.01$).

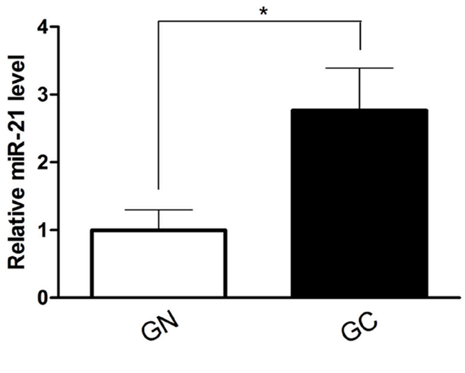

Supplementary Figure S5: Expression levels of miR-21 in human gastric cancer tissues. Quantitative RT-PCR analysis of the expression levels of miR-21 in the same 16 pairs of GC and GN samples (*$P < 0.05$).

Supplementary Table S1: Clinical features of the gastric cancer patients

	Age	Gender	Tumor subtype	Pathological Stage
Case #1	63	male	Adenocarcinoma	IIIA
Case #2	61	male	Adenocarcinoma	IIIA
Case #3	46	female	Signet-ring cell carcinoma	IIIB
Case #4	56	male	Adenocarcinoma	IIIC
Case #5	66	female	Adenocarcinoma	IIIC
Case #6	63	male	Adenocarcinoma	IB
Case #7	58	male	Adenocarcinoma	IIB
Case #8	65	male	Adenocarcinoma	IIB
Case #9	49	male	Adenocarcinoma	IIIC
Case #10	71	female	Adenocarcinoma	IIIC
Case #11	65	female	Adenocarcinoma	IIIB
Case #12	64	male	Adenocarcinoma	IIIA
Case #13	65	male	Adenocarcinoma	IIB
Case #14	58	male	Adenocarcinoma	IIIB
Case #15	70	female	Adenocarcinoma	IIA
Case #16	53	male	Adenocarcinoma	IV
